# A Genetic Variation in the Y Chromosome Among Modern Japanese Males Related to Several Physiological and Psychological Characteristics

**DOI:** 10.3389/fnbeh.2021.774879

**Published:** 2021-12-02

**Authors:** Masahiro Matsunaga, Yohsuke Ohtsubo, Takahiko Masuda, Yasuki Noguchi, Hidenori Yamasue, Keiko Ishii

**Affiliations:** ^1^Department of Health and Psychosocial Medicine, Aichi Medical University School of Medicine, Nagakute, Japan; ^2^Department of Social Psychology, Graduate School of Humanities and Sociology, The University of Tokyo, Bunkyō, Japan; ^3^Department of Psychology, University of Alberta, Edmonton, AB, Canada; ^4^Department of Psychology, Graduate School of Humanities, Kobe University, Kobe, Japan; ^5^Department of Psychiatry, Hamamatsu University School of Medicine, Hamamatsu, Japan; ^6^Department of Cognitive and Psychological Sciences, Graduate School of Informatics, Nagoya University, Nagoya, Japan

**Keywords:** haplogroup D-M55, gene polymorphism (genetics), body mass index - BMI, close friends, gene–gene interaction

## Abstract

Previous studies in population genetics have proposed that the Y-chromosomal (Y-DNA) haplogroup D ancestor likely originated from Africa. The haplogroup D branch next started Out-of-Africa migration, rapidly expanded across Eurasia, and later diversified in East Asia. Y-DNA haplogroup D-M55, one of the branches of haplogroup D, is only found in modern Japanese males, suggesting that individuals with Y-DNA haplogroup D migrated from the Eurasian continent. Based on previous observations, Y-DNA haplogroup D is expected to be associated with some male characteristics including personality. Therefore, this study investigated whether the Y-DNA haplogroup D-M55 is associated with several physiological and psychological characteristics, including exploratory motivation and human relationship-related perception. We recruited Japanese young adult males and females and investigated the association between Y-DNA haplogroup D-M55, physiological [body mass index (BMI)], and several psychological parameters [perceived number of close friends, behavioral inhibition system/behavioral activation system (BIS/BAS), perceived happiness, and perceived loneliness]. The results indicated that males with haplogroup D-M55 had a higher BMI and more close friends, compared with non-carrier males. Additional multiple regression analyses, which tested the hypothesis that haplogroup D-M55 predicts BMI and perceived number of close friends, confirmed our hypothesis, even after controlling for the potentially confounding variables of age and sex. We also analyzed the gene–gene interaction between haplogroup D-M55 and an autosomal gene polymorphism associated with BMI and human relationships, such as the dopamine D2 receptor gene (*DRD2*: rs1800497). Results showed gene–gene interactions between haplogroups D-M55 and *DRD2* in BMI. Based on these findings, it is demonstrated that Y-DNA haplogroup D is associated with human personality.

## Introduction

We inherit half of our DNA from our father and half from our mother. Genetic modification occurs frequently when DNA is transmitted from parents, and even siblings with the same parents have different DNA. However, there are some instances in which the DNA of the parent is inherited with little change. These are maternal mitochondrial DNA and paternal Y-chromosomal DNA (Y-DNA). Because both mitochondrial DNA and Y-DNA change little by little due to mutations, we can assume the order in which the existing mutations were separated from their common ancestor. Analyses of haplogroups and populations of similar haplotypes (haploid genotype) with a common ancestor are widely used in population genetics (Rindermanna et al., 2012). For example, the origin of Japanese people can be explored by examining Y-DNA haplogroups. Y-chromosomal Adam, “a male ancestor common to all human beings,” was estimated to be a male from 200,000 to 300,000 years ago (Karmin et al., 2015). Previous studies have proposed that the Y-DNA haplogroup D ancestor likely lived in Africa around 50,000–70,000 years ago and that the haplogroup D branch (haplogroup D1-M174) started Out-of-Africa migration and rapidly expanded across Eurasia, later diversifying in East Asia (Haber et al., 2019; Hallast et al., 2021). In Japan, haplogroup D-M55 (haplogroup D1a2a: one of two branches of haplogroup D1a) is found in approximately 33% of modern Japanese males (Sato et al., 2014).

The genetic variations in Y-DNA have been reported to influence male behavior, health risk, and immunity. For example, Wang et al. (2012) found a protective effect of Y-haplogroup E1b1b1c against prostate cancer. Furthermore, Dumanski et al. (2015) reported that smoking is associated with the loss of chromosome Y in blood cells, which is associated with an increased risk of non-hematological tumors among aging males. Charchar et al. (2012) also reported that Y-haplogroup I is linked to a substantially increased risk of coronary artery disease, due to the downregulation of adaptive immunity as well as upregulation of inflammatory response pathways in their macrophages. Furthermore, Sato et al. (2013) also found that Y-DNA haplogroup D-M55 is associated with spermatogenic failure in Japanese males. Sermondade et al. (2012) also showed a negative correlation between body mass index (BMI) and sperm concentration or total sperm count, suggesting an association between haplogroup D-M55 and several male physiological parameters, such as BMI. Recently, Mchiza et al. (2019) reported that BMI is associated with social and psychological parameters, such as psychological distress or mental health wellness, suggesting an association between haplogroup D-M55 and several male psychological parameters, such as perceived happiness and loneliness. Based on these previous observations, it is possible that Y-DNA haplogroup D is associated with some male personality traits.

Although Y-DNA haplogroups could be a marker of many environmental and genetic differences and (most probably) might not be genes directly coding the psychological and physiological characteristics, Rindermanna et al. (2012) have indicated that Y-DNA haplogroups could use as evolutionary markers of cognitive ability. In their study, the haplogroups were used in linear multiple regression analysis to determine their impacts as predictors of differences in national intelligence, and haplogroups appeared to be significant predictors of national cognitive ability. Thus, it is possible that Y-DNA haplogroup D is also associated with several cognitive and behavioral traits as previous studies have suggested an association between human exploratory and impulsive behaviors, such as novelty seeking and Out-of-Africa migration (Munafò et al., 2008; Royo et al., 2018). If haplogroup D-M55, a genetic polymorphism unique to Japan, is associated with several behavioral characteristics including exploratory behaviors, it may be deduced that this gene polymorphism is associated with Out-of-Eurasia migration. Therefore, the present study first investigated the association between Y-DNA haplogroup D-M55 and several physiological (BMI) and psychological parameters [perceived number of close friends, behavioral inhibition system/behavioral activation system (BIS/BAS), perceived happiness, and perceived loneliness] in Japanese university students.

Because the biological mechanisms underlying the linkage between haplogroup D-M55 and male personality is still obscure, the association might be on shaky grounding. In order to reinforce the association, we focused on the gene–gene interactions, which can be defined as a logical interaction between two or more genes that affect the phenotype of organisms (Koo et al., 2013; Lee et al., 2013). A study has also analyzed Y chromosome DNA, mitochondrial DNA, and autosomal microsatellite markers together to reveal the evolutionary history of a certain population (Jinam et al., 2012) because these genes are co-evolutional. Thus, it is possible that there is a gene–gene interaction between Y-DNA haplogroup D-M55 and several autosomal gene polymorphisms, and the interaction may induce differences in personality.

This study is a part of our larger research project, an exploratory cross-cultural investigation of various gene × environment effects (for published reports on the project, see: Ishii et al., 2018, 2021; Zheng et al., 2020). We used a subset of the project’s data to test for subtle effects of the haplogroup D-M55 × several autosomal gene polymorphisms on BMI and perceived number of close friends in Japanese university students. In this project, we analyzed several gene polymorphisms, such as serotonin (5-hydroxytryptamine: 5-HT) transporter gene-linked polymorphic region (5-HTTLPR) (Luo et al., 2016), rs806377 (cannabinoid receptor 1 gene: *CNR1*, Matsunaga et al., 2018), rs53576 (oxytocin receptor gene: *OXTR*, Nishina et al., 2015), rs1800497 (dopamine D2 receptor gene: *DRD2*, Takeuchi et al., 2015; Murakami et al., 2017), and rs6311 (5-HT (serotonin) receptor 2A gene: *HTR2A*, Matsunaga et al., 2017). We showed the results of the gene–gene interaction between haplogroups D-M55 and *DRD2* using multiple regression analyses with reference to the study by Erzurumluoglu et al. (2018).

## Materials and Methods

### Participants

We recruited 623 undergraduate students from Nagoya University and Kobe University [292 men and 327 women, and four entries with incomplete gender; mean age (Mage) = 19.60 years, standard deviation (SD) = 1.31, range = 18–25]. The mean BMI of the participants was 20.48 kg/m^2^ (*SD* = 2.26, range: 12.59−34.08). This study was approved by the Ethics Committee of Nagoya University (approval number: NUPSY-190415-M-01) and Kobe University (approval number: 2014-10). All participants volunteered for the study for monetary compensation. They signed an informed consent form prior to participation. After completing the study, they were debriefed and paid for their participation.

A statistical power analysis was conducted using G * Power, version 3.1.9.4 (Faul et al., 2007). We assumed that the effect size of this study would be equivalent to that observed in our previous study (Matsunaga et al., 2018). An *a priori* power analysis estimated the necessary sample size for this study as *N* = 432 (analysis of variance, fixed effects, omnibus, one-way; effect size = 0.15; alpha error = 0.05; 1-beta error = 0.80; number of groups = 3). This study used data from three sub-studies of the aforementioned research project (Matsunaga et al., 2017, 2018; Ishii et al., 2018; Zheng et al., 2020). The first sub-study, which was conducted at Kobe University in 2015, involved 213 participants. The second sub-study, which was conducted at Kobe University in 2018, involved 203 participants. The third sub-study, which was conducted at Nagoya University in 2019, involved 207 participants. Therefore, the dataset analyzed in this study included a total of 623 participants.

### Genotyping

Nail samples were collected, and from these samples, genomic DNA was extracted using ISOHAIR kits (Nippon Gene Co., Ltd., Tokyo, Japan). The Y-DNA Haplogroup D-M55 was amplified by polymerase chain reaction (PCR) using the primers 5’- GTAGGCGTTTGACAGCAGTT and 5’- ACTGGATGACTGATGAAAAGGT (Kumagai et al., 2010; Sato et al., 2014), in a total volume of 25 μl solution containing 100 ng of genomic DNA, 0.4 dNTPs, 0.2 μM of each primer, 1.25 U of Takara LA Taq polymerase (Takara Bio Inc., Shiga, Japan), and GC buffer I (Takara Bio Inc.). An initial denaturation at 95°C for 5 min was followed by 40 cycles of denaturation at 95°C for 30 s, annealing at 53°C for 30 s, extension at 72°C for 1 min, and a final extension at 72°C for 5 min.

In addition, the SNP markers for rs1800497 (*DRD2*) were genotyped using TaqMan^®^ SNP Genotyping Assays (Thermo Fisher Scientific Inc., Waltham, MA, USA), which were functionally tested by the manufacturer and made available on demand. The SNP assay contained forward and reverse PCR primers, as well as two allele-specific probes conjugated with either VIC or FAM fluorescent markers. Each PCR mixture consisted of a DNA template, SNP-specific genotyping assay, and Taqman Genotype master mix (Thermo Fisher Scientific Inc.). All PCRs and allelic discrimination reactions were performed using the StepOne Plus^TM^ Real-Time PCR System (Thermo Fisher Scientific Inc.).

### Evaluation of Several Demographic and Psychological Parameters

The participants were asked to report their gender, age, height, and weight. BMI was calculated using height and weight. They were also asked to report their perceived number of close friends, BIS/BAS (Carver and White, 1994; Takahashi et al., 2007), perceived happiness (Lyubomirsky and Lepper, 1999; Shimai et al., 2004), and perceived loneliness (Russell et al., 1980; Moroi, 1992). The participants’ perceived happiness level was evaluated using the Japanese version of the Subjective Happiness Scale (SHS; Shimai et al., 2004) consisting of four items (e.g., “In general, what is your level of happiness?”) that were rated on a 7-point Likert scale from 1 = “not happy at all” to 7 = “very happy.” These four items were averaged to obtain a single score for subjective happiness (Cronbach’s α coefficient = 0.84). The participant’s perceived loneliness level was evaluated by using the Japanese version of the UCLA Loneliness Scale (Moroi, 1992) consisting of 20 items (e.g., “There is no one I can turn to”) that were rated on a 4-point Likert scale from 1 = “never feel like this” to 4 = “frequently feel like this.” The 20 items were averaged to obtain a single score for perceived loneliness (Cronbach’s α coefficient = 0.92). The BIS/BAS scales are presented as a self-report questionnaire that assesses individual differences in personality dimensions that reflect the sensitivity of two motivational systems: the aversive and appetitive system (BIS and BAS; Gray, 1987; Carver and White, 1994). BAS is believed to regulate appetitive motives, in which the goal is to move toward something that is desired. BIS is said to regulate aversive motives, in which the goal is to move away from something that is unpleasant. The Japanese version of the BIS/BAS scales (Takahashi et al., 2007) consists of 20 items divided into two primary scales: the Behavioral Inhibition System scale (BIS; 7 items) and the Behavioral Activation System scale (BAS; 13 items).

### Statistical Analyses

All data analyses were conducted using SPSS version 27 [International Business Machines Corporation (IBM), Armonk, NY]. Demographic (age and BMI) and psychological (number of close friends, happiness, and loneliness) parameters were analyzed using a one-way (males with haplogroup D-M55, males without haplogroup D-M55, and female) analysis of variance (ANOVA) followed by Bonferroni-corrected multiple comparisons. As it is not possible to determine which is higher or lower by comparing only the two groups of males, we included the female participants as a control group in the comparison. Furthermore, to reveal the gene–gene interaction between haplogroup D-M55 and rs1800497 (*DRD2*), we performed multiple regression analyses to identify potentially confounding variables of age and sex, and the interactions with haplogroups D-M55 and *DRD2*. In the regression analyses, gene polymorphisms and sex were converted into dummy variables and analyzed. Haplogroup D-M55: females = 0, non-carrier males = 1, carrier males = 2; *DRD2*: GG = 0, AG = 1. AA = 2. Sex: female = 0, male = 1.

## Results

### Effects of Y-DNA Haplogroup D-M55 on Demographic and Psychological Characteristics

We first determined haplogroup frequency. The number of males with haplogroup D-M55 was 88 (30.66%) in the present Japanese sample (199 non-carriers and five unidentified participants), which was similar to a previous study (Sato et al., 2014).

Table 1 summarizes the effects of the Y-DNA haplogroup D-M55 on physiological and psychological parameters. A one-way ANOVA revealed a significant main effect of the group (males with haplogroup D-M55, males without haplogroup D-M55, and females) on BMI [*F*_(2,608)_ = 9.53, *p* < 0.01, $\eta_{p}^{2}$ = 0.030], perceived number of close friends [*F*_(2,606)_ = 6.19, *p* < 0.01, $\eta_{p}^{2}$ = 0.020], BIS [*F*_(2,577)_ = 9.68, *p* < 0.01, $\eta_{p}^{2}$ = 0.032], subjective happiness [*F*_(2,611)_ = 3.84, *p* = 0.022, $\eta_{p}^{2}$ = 0.012], and loneliness [*F*_(2,610)_ = 3.93, *p* = 0.020, $\eta_{p}^{2}$ = 0.013]. There were no significant differences in age and BAS.

**Table 1:** Effects of haplogroup D-M55 on demographic and psychological parameters.

	Males with haplogroup D	Males without haplogroup D	Females	*F*-value	*p*-value
Age	19.74 (1.25)	19.66 (1.22)	19.53 (1.37)	1.244	0.289
BMI	21.35 (2.36)	20.57 (2.51)	20.19 (2.01)	9.534	<0.01
Number of close friends	11.26 (16.44)	8.228 (8.46)	7.201 (7.56)	6.195	<0.01
BIS	2.97 (0.57)	3.01 (0.58)	3.20 (0.56)	9.687	<0.01
BAS	3.01 (0.43)	3.05 (0.42)	3.05 (0.41)	0.369	0.692
Happiness	4.651 (1.03)	4.560 (1.07)	4.821 (1.08)	3.846	0.022
Loneliness	1.894 (0.48)	2.006 (0.50)	1.886 (0.49)	3.936	0.020

*Note: Results are expressed as means and standard deviations. The variables were compared using a one-way ANOVA followed by Bonferroni-corrected multiple comparisons, and the *F*-value and *p*-value of ANOVA are shown*.

**Figure 1:**
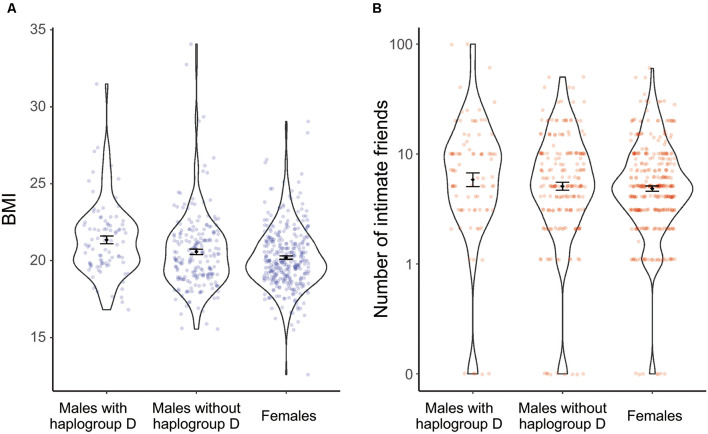
Effects of haplogroup D-M55 on the BMI **(A)** and perceived number of close friends **(B)**. Each column and its error bars represent the mean ± standard error of the mean.

Multiple comparisons showed that the BMI of males with haplogroup D-M55 was significantly higher than that of non-carrier males (*p* = 0.020) and that of females (*p* < 0.01). The BMI of non-carrier males was not significantly different from that of females (*p* = 0.176; Figure 1).

As for the number of close friends, males with haplogroup D-M55 had more friends than non-carrier males (*p* = 0.042) and females (*p* < 0.01). The number of close friends of non-carrier males was not significantly different from that of females (*p* = 0.711; Figure 1). Thus, haplogroup D-M55 in males was associated with a higher BMI and more close friends, compared with non-carrier males and females.

Considering other psychological parameters such as BIS, subjective happiness, and loneliness, females had significantly higher BIS scores than both males with haplogroup D-M55 (*p* < 0.01) and non-carrier males (*p* < 0.01), whereas there was no significant difference between the male groups (*p* = 1.000). The mean SHS score in females was higher than in non-carrier males (*p* = 0.021), whereas the differences between females and males with haplogroup D-M55 (*p* = 0.553), and between males with haplogroup D-M55 and non-carrier males (*p* = 1.000) were not significant. The mean score for the Loneliness Scale among non-carrier males was significantly higher than for females (*p* = 0.020), whereas the difference between females and males with haplogroup D-M55 (*p* = 1.000), and between males with haplogroup D-M55 and non-carrier males (*p* = 0.224) were not significant.

### Gene–Gene Interaction Between Y-DNA Haplogroup D-M55 and Dopamine D2 Receptor Polymorphism

We subsequently performed a multiple regression analysis, which tested our hypothesis that haplogroup D-M55 predicted BMI and the number of close friends, even after controlling for the potentially confounding variables of age and sex. Table 2 (step 1) shows the results of the multiple regression analysis for BMI. The model was statistically significant [*F*_(3,607)_ = 6.363, *p* < 0.01], supporting our hypothesis (*β* = 0.248, *t* = 2.708, *p* < 0.01). Furthermore, to explore the gene–gene interaction between Y-DNA haplogroup D-M55 and an autosomal SNP associated with BMI, we performed a supplementary analysis of BMI, haplogroup D-M55, and *DRD2* (rs1800497). Table 2 (step 2) shows the results of the multiple regression analysis for BMI entering the interactions with haplogroups D-M55 and *DRD2*. The model was statistically significant [*F*_(5,597)_ = 5.199, *p* < 0.01], and gene–gene interaction was also statistically significant (*β* = 0.101, *t* = 2.484, *p* = 0.013). The average BMIs of each group are presented in Table 3.

**Table 2:** Results from the regression analysis examining the association between Y-DNA haplogroup D-M55 and BMI.

	Step 1			Step 2		
Predictor variables	*β*	*t*	*p*-value	*β*	*t*	*p*-value
**Haplogroup D-M55**	**0.248**	** 2.708**	< 0.01	**0.205**	**2.187**	**0.029**
Sex	−0.087−	−0.953−	0.341	−0.052−	−0.560−	0.575
Age	0.009	0.230	0.818	0.004	0.108	0.914
*DRD2*				0.032	0.791	0.429
**Haplogroup D-M55** × *DRD2*				**0.101**	**2.484**	**0.013**
Adjusted *R*^2^	0.026			0.034		

*Note: All predictor variables were included in the regression analysis. Boldface indicates the statistically significant variables. *β*, standardized beta coefficient; BMI, body mass index*.

**Table 3:** BMI of each group.

*DRD2* Genotype	Males with haplogroup D	Males without haplogroup D	Females
AA	22.21 (2.37)	20.91 (2.10)	19.79 (1.62)
AG	21.26 (2.15)	20.64 (2.88)	20.23 (1.99)
GG	20.75 (2.39)	20.32 (2.27)	20.27 (2.14)

*Note: Results are expressed as means and standard deviations*.

Table 4 (step 1) shows the results of the multiple regression analysis for the perceived number of close friends, after controlling for the potentially confounding variables of age and sex. The model was statistically significant [*F*_(3,605)_ = 4.941, *p* < 0.01], supporting our hypothesis (*β* = 0.230, *t* = 2.489, *p* < 0.05). Table 4 (step 2) shows the results of the multiple regression analysis for the perceived number of close friends entering the interactions with haplogroups D-M55 and *DRD2*. The model was statistically significant [*F*_(5,595)_ = 3.033, *p* = 0.010]; however, the gene–gene interaction was not statistically significant.

**Table 4:** Results from the regression analysis examining the association between Y-DNA haplogroup D-M55 and the perceived number of close friends.

	Step 1			Step 2		
Predictor variables	*β*	*t*	*p*-value	*β*	*t*	*p*-value
**Haplogroup D-M55**	**0.230**	**2.489**	**0.013**	**0.225**	**2.375**	**0.018**
Sex	−0.101−	−1.099−	0.272	−0.096−	−1.025−	0.306
Age	−0.062−	−1.550−	0.122	−0.064−	−1.568−	0.117
*DRD2*				0.014	−0.337−	0.736
Haplogroup D-M55 × *DRD2*				0.012	−0.288−	0.773
Adjusted *R*^2^	0.019			0.017		

*Note: All predictor variables were included in the regression analysis. Boldface indicates the statistically significant variables. *β*: Standardized beta coefficient*.

## Discussion

### General Discussion

Based on previous studies indicating that Y-DNA haplogroup is associated with some male personalities (Sato et al., 2013; Dumanski et al., 2015), this study posits that Y-DNA haplogroup D-M55, a genetic polymorphism unique to Japan, may be associated with certain personality traits. In the present study, we investigated the association between haplogroup D-M55 and several physiological and psychological parameters, including BMI, number of close friends, BIS/BAS, subjective happiness, and loneliness. As shown in Table 1, our statistical analyses indicated that males with haplogroup D-M55 had higher BMI and more close friends than both non-carrier males and females. Friendships are adaptive (Seyfarth and Cheney, 2012), and having a strong social network reduces stress, lowers the risk of disease, and increases longevity (Berkman et al., 2004; Holt-Lunstad et al., 2010). Thus, from the perspective of evolutionary psychology, it seems that individuals with higher BMI and many friends have an advantage in terms of survival. Hence, it is possible that these features may be one of the factors associated with human survival or Out-of-Eurasia migration.

However, both males with and without haplogroup D-M55 have normal BMI; hence, the results are difficult to directly interpret with respect to survival and migration. As previous studies have suggested an association between human exploratory and impulsive behaviors, such as novelty seeking and Out-of-Africa migration (Munafò et al., 2008; Royo et al., 2018), we subsequently examined the association between haplogroup D-M55 and human motivational systems such as the aversive or behavioral inhibition system (BIS) and the appetitive or behavioral activation system (BAS). However, we did not find a significant difference in BIS/BAS scores between males with haplogroup D-M55 and non-carrier males. In addition, we did not find a difference in either happiness or loneliness scores between males with haplogroup D-M55 and non-carrier males. However, males without haplogroup D-M55 were significantly lonelier than females, whereas males with haplogroup D-M55 were at the same level of loneliness as females. Thus, it is possible that a significant difference between males was not observed due to the small sample size.

Although the biological mechanisms underlying the association between Y-DNA haplogroup D-M55, BMI, and perceived number of close friends are still obscure, previous reports have indicated an association between infertility and Y-DNA haplogroup D-M55 in Japanese males (Sato et al., 2013). Several previous reports have also shown a negative correlation between BMI and sperm concentration or total sperm count (Sermondade et al., 2012). Thus, the present finding, indicating the association between BMI and haplogroup D-M55, might be valid. A recent study has demonstrated the elevated expression of cytochrome P450 aromatase, which catalyzes the conversion of testosterone to estradiol in Leydig cells of men with nonobstructive azoospermia, and elevated circulating levels of luteinizing hormone (LH) and follicle-stimulating hormone (FSH), which induce aromatase expression (Shiraishi et al., 2021). A negative correlation between circulating LH level and BMI, and a negative correlation between LH and testosterone have been previously reported (Pagán et al., 2006; Fukui et al., 2007). Testosterone is known to be associated with aggressive behavior (Carré and Archer, 2018). Therefore, it is suggested that males with haplogroup D-M55 have lower circulating testosterone levels and are less aggressive. As a result, it is possible for them to build good relationships with friends. Further studies measuring the circulating levels of steroid hormones and gonadotropins in males with haplogroup D-M55 are needed in the future.

To investigate the gene–gene interactions between Y-DNA haplogroup D-M55 and autosomal genetic polymorphisms associated with BMI and human relationships, we performed a supplementary analysis of the interactions with haplogroup D-M55 and autosomal genetic polymorphism (*DRD2*). The results showed gene–gene interactions between haplogroups D-M55 and *DRD2* in BMI. A previous study indicated that there was a significant association between the *DRD2* genotype and exercise habit in the period from childhood to adolescence, with a 1.38-fold greater probability of people who exercise carrying the A allele among Japanese adults (Murakami et al., 2017). The rs1800497 pathogenic variant (A allele) has been previously described to be associated with a reduction in *DRD2* receptor density in the brain (Comings et al., 1991); moreover, *DRD2* A allele carriers experienced greater positive emotions after cocaine exposure (Spellicy et al., 2014), suggesting that individuals with *DRD2* A allele experience a lower level of “reward” due to reduced dopaminergic functions and are therefore more habitual in seeking “artificial” means of increasing the “reward” sensation, such as exercise. Moreover, previous studies have indicated that *DRD2* polymorphism is associated with feelings of integration or closeness to their local community (Pearce et al., 2017, 2018), suggesting that variation in genes associated with dopamine receptor 2 may be predominantly linked to engagement in wider social groups, resulting in higher motivation for several behaviors, including exercise. Although the biological mechanisms underlying the association between haplogroup D-M55 genotype and *DRD2* remain unclear, it is possible that the dopaminergic nervous system is involved in these biological mechanisms.

### Limitations and Future Directions

Our study has several limitations. First, in the present study, we did not consider some basic demographics, such as the disease states, medication intake, and socioeconomic status (SES), between the three groups. In the case of the present study, the SES may represent a confounding variable. Because a previous study reported that lower-class individuals are higher in empathic accuracy than are upper-class individuals (Kraus et al., 2010), it may influence the number of close friends. Thus, several confounding factors may exist in the present results. Second, because height and weight were self-reported, it might differ from actual BMI. Third, in the present study, we used only the BIS/BAS questionnaire to investigate the association between haplogroup D-M55 and the human motivational system. Previous studies have used many experimental methods to examine the association between gene polymorphisms and human exploratory and impulsive behaviors, such as the human behavioral pattern monitor (hBPM), an exploration paradigm based on the rodent open field (Minassian et al., 2018), and the Go/No-go task (Nomura and Nomura, 2006). Thus, we cannot confidently say that haplogroup D-M55 is not associated with human exploratory and impulsive behaviors. Fourth, the sample size of the present study may be relatively small because the number of males with haplogroup D-M55 was small in the Japanese samples. Thus, replicating the present experiment using larger samples is necessary to draw stronger conclusions. Fifth, considering the gene–gene interaction, the present study investigated the interaction between Y-DNA haplogroup D-M55 and not only *DRD2*, but also other autosomal gene polymorphisms associated with BMI and human relationships, such as 5HTTLPR (Luo et al., 2016), rs806377 (*CNR1*; Matsunaga et al., 2018), rs53576 (*OXTR*; Nishina et al., 2015), and rs6311 (*HTR2A*; Matsunaga et al., 2017). However, we did not mention these interactions in this article. We were unable to find any association other than *DRD2* and haplogroup D-M55 (data not shown). In addition to *DRD2*, there are many SNPs related to BMI and human relationships; therefore, it is necessary to investigate the relationships with other SNPs in the future. For example, the genome-wide association study (GWAS) has recently been developed to search the candidate genes comprehensively, e.g., BMI (Akiyama et al., 2017), and antisocial behavior (Tielbeek et al., 2017). Thus, comparing the SNPs of males with and without haplogroup D-M55 comprehensively by GWAS, it is possible to search for candidate genes related to gene–gene interactions other than *DRD2*. Furthermore, recent studies have suggested an association between personality-associated genes and Out-of-Africa migration (Chen et al., 1999; Royo et al., 2018). It is known that exon 3 variable-number tandem repeat polymorphism in the dopamine D4 receptor gene (*DRD4*) is associated with human exploratory behavior, such as novelty seeking (Munafò et al., 2008), and a previous study found a correlation between the distance of the macro-migration in nomadic populations and the proportion of long alleles of *DRD4*, which is expected to be strongly associated with novelty seeking (Chen et al., 1999). A previous report also found a correlation between the migration distance and allele frequency of copy-number variants within the signal regulatory protein beta-1 (SIRPB1) gene, which is associated with impulsive behavior (Royo et al., 2018). Such gene polymorphisms might also be the candidate genes related to gene–gene interactions.

### Conclusion

The present study is the first to show the effects of haplogroup D-M55 on psychological and physiological characteristics. Our findings indicate that males with haplogroup D-M55 are characterized as having a higher BMI and many close friends. These findings demonstrate that Y-DNA haplogroup D is associated with human personality. The present findings may be applicable to the fields of evolutionary psychology and population genetics.

## Data Availability Statement

The original contributions presented in the study are included in the article, further inquiries can be directed to the corresponding author.

## Ethics Statement

The studies involving human participants were reviewed and approved by Nagoya University and Kobe University. Written informed consent was obtained from all participants for their participation in this study.

## Author Contributions

MM, YO, TM, YN, HY, and KI designed the research. YO, TM, YN, and KI collected the data. MM determined the gene polymorphisms and analyzed the data. MM, YO, and KI drafted the manuscript. TM, YN, and HY provided critical revisions. All authors contributed to the article and approved the submitted version.

## Conflict of Interest

The authors declare that the research was conducted in the absence of any commercial or financial relationships that could be construed as a potential conflict of interest.

## Publisher’s Note

All claims expressed in this article are solely those of the authors and do not necessarily represent those of their affiliated organizations, or those of the publisher, the editors and the reviewers. Any product that may be evaluated in this article, or claim that may be made by its manufacturer, is not guaranteed or endorsed by the publisher.

## References

[B1] Akiyama M. Okada Y. Kanai M. Takahashi A. Momozawa Y. Ikeda M.. (2017). Genome-wide association study identifies 112 new loci for body mass index in the Japanese population. Nat. Genet. 49, 1458–1467. 10.1038/ng.3951 28892062

[B2] Berkman L. F. Melchior M. Chastang J. F. Niedhammer I. Leclerc A. Goldberg M. (2004). Social integration and mortality: a prospective study of French employees of Electricity of France-Gas of France: the GAZEL cohort. Am. J. Epidemiol. 159, 167–174. 10.1093/aje/kwh020 14718219

[B3] Carré J. M. Archer J. (2018). Testosterone and human behavior: the role of individual and contextual variables. Curr. Opin. Psychol. 19, 149–153. 10.1016/j.copsyc.2017.03.021 29279215

[B4] Carver C. S. White T. L. (1994). Behavioral inhibition, behavioral activation and affective responses to impending reward and punishment: the BIS/BAS scales. J. Pers. Soc. Psychol. 67, 319–333. 10.1037/0022-3514.67.2.319

[B5] Charchar F. J. Bloomer L. D. Barnes T. A. Cowley M. J. Nelson C. P. Wang Y.. (2012). Inheritance of coronary artery disease in men: an analysis of the role of the Y chromosome. Lancet 379, 915–922. 10.1016/S0140-6736(11)61453-0 22325189 PMC3314981

[B6] Chen C. S. Burton M. Greenberger E. Dmitrieva J. (1999). Population migration and the variation of dopamine D4 receptor (DRD4) allele frequencies around the globe. Evol. Hum. Behav. 20, 309–324. 10.1016/S1090-5138(99)00015-X

[B7] Comings D. E. Comings B. G. Muhleman D. Dietz G. Shahbahrami B. Tast D.. (1991). The dopamine D2 receptor locus as a modifying gene in neuropsychiatric disorders. JAMA 266, 1793–1800. 1832466

[B8] Dumanski J. P. Rasi C. Lönn M. Davies H. Ingelsson M. Giedraitis V.. (2015). Mutagenesis. Smoking is associated with mosaic loss of chromosome Y. Science 347, 81–83. 10.1126/science.1262092 25477213 PMC4356728

[B9] Erzurumluoglu A. M. Baird D. Richardson T. G. Timpson N. J. Rodriguez S. (2018). Using Y-chromosomal haplogroups in genetic association studies and suggested implications. Genes (Basel) 9:45. 10.3390/genes9010045 29361760 PMC5793196

[B10] Faul F. Erdfelder E. Lang A. G. Buchner A. (2007). G*Power 3: a flexible statistical power analysis program for the social, behavioral and biomedical sciences. Behav. Res. Methods 39, 175–191. 10.3758/bf03193146 17695343

[B11] Fukui M. Soh J. Tanaka M. Kitagawa Y. Hasegawa G. Yoshikawa T.. (2007). Low serum testosterone concentration in middle-aged men with type 2 diabetes. Endocr. J. 54, 871–877. 10.1507/endocrj.k07-077 17998764

[B12] Gray J. A. (1987). The Psychology of Fear and Stress. Cambridge: Cambridge University Press.

[B13] Haber M. Jones A. L. Connell B. A. Asan A. E. Yang H. Thomas M. G.. (2019). A rare deep-rooting D0 african Y-chromosomal haplogroup and its implications for the expansion of modern humans out of africa. Genetics 212, 1421–1428. 10.1534/genetics.119.302368 31196864 PMC6707464

[B14] Hallast P. Agdzhoyan A. Balanovsky O. Xue Y. Tyler-Smith C. (2021). A Southeast Asian origin for present-day non-African human Y chromosomes. Hum. Genet. 140, 299–307. 10.1007/s00439-020-02204-9 32666166 PMC7864842

[B15] Holt-Lunstad J. Smith T. B. Layton JB. (2010). Social relationships and mortality risk: a meta-analytic review. PLoS Med. 7:e1000316. 10.1371/journal.pmed.1000316 20668659 PMC2910600

[B16] Ishii K. Matsunaga M. Noguchi Y. Yamasue H. Ochi M. Ohtsubo Y. (2018). A polymorphism of serotonin 2A receptor (5-HT2AR) influences delay discounting. Pers. Individ. Dif. 121, 193–199. 10.1016/j.paid.2017.03.011

[B014] Ishii K. Masuda M. Matsunaga M. Noguchi Y. Yamasue H. Ohtsubo Y. (2021). A reexamination of the effects of culture and dopamine D4 receptor gene interaction on social orientation. Psychologia 10.2117/psysoc.2021-B014

[B17] Jinam T. A. Hong L. C. Phipps M. E. Stoneking M. Ameen M. Edo J.. (2012). Evolutionary history of continental southeast asians: “early train” hypothesis based on genetic analysis of mitochondrial and autosomal DNA Data. Mol. Biol. Evol. 29, 3513–3527. 10.1093/molbev/mss169 22729749

[B18] Karmin M. Saag L. Vicente M. Sayres M. A. W. Järve M. Talas U. G.. (2015). A recent bottleneck of Y chromosome diversity coincides with a global change in culture. Genome Res. 25, 459–466. 10.1101/gr.186684.114 25770088 PMC4381518

[B19] Koo C. L. Liew M. J. Mohamad M. S. Salleh A. H. (2013). A review for detecting gene–gene interactions using machine learning methods in genetic epidemiology. Biomed Res. Int. 2013:432375. 10.1155/2013/432375 24228248 PMC3818807

[B20] Kraus M. W. Côté S. Keltner D. (2010). Social class, contextualism and empathic accuracy. Psychol. Sci. 21, 1716–1723. 10.1177/0956797610387613 20974714

[B21] Kumagai R. Sasaki Y. Tokuta T. Biwasaka H. Matsusue A. Aoki Y.. (2010). Distinct breakpoints in two cases with deletion in the Yp11.2 region in Japanese population. Hum. Genet. 127, 537–543. 10.1007/s00439-010-0794-7 20127364

[B22] Lee Y. Ghosh D. Zhang Y. (2013). Association testing to detect gene–gene interactions on sex chromosomes in trio data. Front. Genet. 13:239. 10.3389/fgene.2013.00239 24312118 PMC3826485

[B23] Luo S. Yu D. Han S. (2016). Genetic and neural correlates of romantic relationship satisfaction. Soc. Cogn. Affect. Neurosci. 11, 337–348. 10.1093/scan/nsv117 26385612 PMC4733345

[B24] Lyubomirsky S. Lepper H. S. (1999). A measure of subjective happiness: preliminary reliability and construct validation. Soc. Indicators Res. 46, 137–155. 10.1023/A:1006824100041

[B25] Matsunaga M. Kawamichi H. Umemura T. Hori R. Shibata E. Kobayashi F.. (2017). Neural and genetic correlates of the social sharing of happiness. Front. Neurosci. 11:718. 10.3389/fnins.2017.00718 29311795 PMC5742108

[B26] Matsunaga M. Masuda T. Ishii K. Ohtsubo Y. Noguchi Y. Ochi M.. (2018). Culture and cannabinoid receptor gene polymorphism interact to influence the perception of happiness. PLoS One 13:e0209552. 10.1371/journal.pone.0209552 30576341 PMC6303049

[B27] Mchiza Z. J.-R. Parker W. A. Hossin M. Z. Heshmati A. Labadarios D. Falkstedt D.. (2019). Social and psychological predictors of body mass index among south africans 15 years and older: SANHANES-1. Int. J. Environ. Res. Public Health 16:3919. 10.3390/ijerph16203919 31618952 PMC6843690

[B28] Minassian A. Young J. W. Geyer M. A. Kelsoe J. R. Perry W. (2018). The COMT Val158Met polymorphism and exploratory behavior in bipolar mania. Mol. Neuropsychiatry 3, 151–156. 10.1159/000481822 29594134 PMC5836247

[B29] Moroi K. (1992). Dimensions of the Revised UCLA Loneliness Scale’s dimensions. Shizuoka Univ. Repos. 42, 23–51. 10.14945/00003890

[B30] Munafò M. R. Yalcin B. Willis-Owen S. A. Flint J. (2008). Association of the dopamine D4 receptor (DRD4) gene and approach-related personality traits: meta-analysis and new data. Biol. Psychiatry 63, 197–206. 10.2147/OPTH.S332565 17574217

[B31] Murakami H. Fuku N. Kawakami R. Gando Y. Iemitsu M. Sanada K.. (2017). Retraction Notice to: DRD2/ANKK1 gene polymorphism rs1800497 is associated with exercise habit in the period from childhood to adolescence in Japanese. JPFSM 6, 95–102. 10.7600/jpfsm.6.95

[B32] Nishina K. Takagishi H. Inoue-Murayama M. Takahashi H. Yamagishi T. (2015). Polymorphism of the oxytocin receptor gene modulates behavioral and attitudinal trust among men but not women. PLoS One 10:e0137089. 10.1371/journal.pone.0137089 26444016 PMC4621758

[B33] Nomura M. Nomura Y. (2006). Psychological, neuroimaging and biochemical studies on functional association between impulsive behavior and the 5-HT2A receptor gene polymorphism in humans. Ann. N. Y. Acad. Sci. 1086, 134–143. 10.1196/annals.1377.004 17185512

[B34] Pagán Y. L. Srouji S. S. Jimenez Y. Emerson A. Gill S. Hall J. E. (2006). Inverse relationship between luteinizing hormone and body mass index in polycystic ovarian syndrome: investigation of hypothalamic and pituitary contributions. J. Clin. Endocrinol. Metab. 91, 1309–1316. 10.1210/jc.2005-2099 16434454

[B36] Pearce E. Wlodarski R. Machin A. Dunbar R. I. M. (2017). Variation in the β-endorphin, oxytocin and dopamine receptor genes is associated with different dimensions of human sociality. Proc. Natl. Acad. Sci. U S A 114, 5300–5305. 10.1073/pnas.1700712114 28461468 PMC5441808

[B35] Pearce E. Wlodarski R. Machin A. Dunbar R. I. M. (2018). The influence of genetic variation on social disposition, romantic relationships and social networks: a replication study. Adapt. Hum. Behav. Physiol. 4, 400–422. 10.1007/s40750-018-0101-8 30393594 PMC6190642

[B37] Rindermanna H. Woodley M. A. Stratford J. (2012). Haplogroups as evolutionary markers of cognitive ability. Intelligence 40, 362–375. 10.1016/j.intell.2012.04.002

[B38] Royo J. L. Valls J. Acemel R. D. Gómez-Marin C. Pascual-Pons M. Lupiañez A.. (2018). A common copy-number variant within SIRPB1 correlates with human Out-of-Africa migration after genetic drift correction. PLoS One 13:e0193614. 10.1371/journal.pone.0193614 29518122 PMC5843225

[B39] Russell D. Peplau L. A. Cutrona C. E. (1980). The revised UCLA Loneliness Scale: Concurrent and discriminant validity evidence. J. Pers. Soc. Psychol. 39, 472–480. 10.1037//0022-3514.39.3.472 7431205

[B40] Sato Y. Shinka T. Ewis A. A. Yamauchi A. Iwamoto T. Nakahori Y. (2014). Overview of genetic variation in the Y chromosome of modern Japanese males. Anthropol. Sci. 122, 131–136. 10.1537/ase.140709

[B41] Sato Y. Shinka T. Iwamoto T. Yamauchi A. Nakahori Y. (2013). Y chromosome haplogroup D2* lineage is associated with azoospermia in Japanese males. Biol. Reprod. 88:107. 10.1095/biolreprod.112.105718 23467741

[B42] Sermondade N. Faure C. Fezeu L. Lévy R. Czernichow S. (2012). Obesity-fertility collaborative group. Obesity and increased risk for oligozoospermia and azoospermia. Arch. Int. Med. 172, 440–442. 10.1001/archinternmed.2011.1382 22412113 PMC5151180

[B43] Seyfarth R. M. Cheney D. L. (2012). The evolutionary origins of friendship. Annu. Rev. Psychol. 63, 153–177. 10.1146/annurev-psych-120710-100337 21740224

[B44] Shimai S. Otake K. Utsuki N. Ikemi A. Lyubomirsky S. (2004). Development of a Japanese version of the subjective happiness scale (SHS) and examination of its validity and reliability. Nihon Koshu Eisei zasshi 51, 845–853. 10.11236/jph.51.10_845 15565993

[B45] Shiraishi K. Oka S. Matsuyama H. (2021). Testicular testosterone and estradiol concentrations and aromatase expression in men with nonobstructive azoospermia. J. Clin. Endocrinol. Metab. 106, e1803–e1815. 10.1210/clinem/dgaa860 33236081

[B46] Spellicy C. J. Harding M. J. Hamon S. C. Mahoney J. J. Reyes J. A. Kosten T. R.. (2014). A variant in ANKK1 modulates acute subjective effects of cocaine: a preliminary study. Genes Brain Behav. 13, 559–564. 10.1111/gbb.12121 24528631 PMC4079840

[B47] Takahashi Y. Yamagata S. Kijima N. Shigemasu K. Ono Y. Ando J. (2007). Gray’s temperament model: development of Japanese version of BIS/BAS scales and a behavior genetic investigation using the twin method. Jpn. J. Pers. 15, 276–289. 10.2132/personality.15.276

[B48] Takeuchi H. Tomita H. Taki Y. Kikuchi Y. Ono C. Yu Z.. (2015). The associations among the dopamine D2 receptor Taq1, emotional intelligence, creative potential measured by divergent thinking and motivational state and these associations’ sex differences. Front. Psychol. 6:912. 10.3389/fpsyg.2015.00912 26217259 PMC4493369

[B49] Tielbeek J. J. Johansson A. Polderman T. J. C. Rautiainen M. R. Jansen P. Taylor M.. (2017). Genome-wide association studies of a broad spectrum of antisocial behavior. JAMA Psychiatry. 74, 1242–1250. 10.1001/jamapsychiatry.2017.3069 28979981 PMC6309228

[B50] Wang Z. Parikh H. Jia J. Myers T. Yeager M. Jacobs K. B.. (2012). Y chromosome haplogroups and prostate cancer in populations of European and Ashkenazi Jewish ancestry. Hum. Genet. 131, 1173–1185. 10.1007/s00439-012-1139-5 22271044 PMC3374121

[B51] Zheng S. Masuda T. Matsunaga M. Noguchi Y. Ohtsubo Y. Yamasue H.. (2020). Oxytocin receptor gene (OXTR) and childhood adversity influence trust. Psychoneuroendocrinology 121:104840. 10.1016/j.psyneuen.2020.104840 32866773

